# Furosemide Stress Test and Renal Resistive Index for Prediction of Severity of Acute Kidney Injury in Sepsis

**DOI:** 10.7759/cureus.44408

**Published:** 2023-08-30

**Authors:** Pravin K Das, Sudeep K Maurya, Soumya Sankar Nath, Tushant Kumar, Namrata Rao, Neha Shrivastava

**Affiliations:** 1 Anaesthesiology and Critical Care Medicine, Dr. Ram Manohar Lohia Institute of Medical Sciences, Lucknow, IND; 2 Anaesthesiology, Dr. Ram Manohar Lohia Institute of Medical Sciences, Lucknow, IND; 3 Radiodiagnosis, Dr. Ram Manohar Lohia Institute of Medical Sciences, Lucknow, IND; 4 Nephrology, Dr. Ram Manohar Lohia Institute of Medical Sciences, Lucknow, IND; 5 Anaesthesiology, Medipark Hospital, Patna, IND

**Keywords:** furosemide stress test, severe sepsis, renal replacement therapy (rrt), : acute kidney injury, renal resistive index

## Abstract

Introduction

The furosemide stress test (FST) predicts the severity and the need for renal replacement therapy (RRT) in patients with sepsis-associated acute kidney injury (S-AKI). The renal resistive index (RRI) indicates renal vascular resistance.

Objectives

The primary objective was to find the correlation between FST and RRI in S-AKI. The secondary objectives were to evaluate the role of FST and RRI on the progression of S-AKI.

Methods

A total of 154 consenting adult patients with S-AKI were administered FST. Renal echography was performed within the first 12 hours of admission, and RRI was calculated. The patients were grouped either into progressors or non-progressors to AKI-KDIGO stage 3.

Results

Of the patients who had RRI at Day 1 less than 0.73, 60% recovered, 34.3% needed RRT, and 35.5% died, whereas in those who had RRI at Day 1 greater than 0.73, only 22% recovered, 46.6% required RRT, and 51.6% died. RRI value of 0.73 predicted the need for RRT with a sensitivity of 35.1%, specificity of 80.4% and accuracy of 69.1%. The highest number of patients of KDIGO stage 3 (50%), followed by stage 2 (28.1%) and stage 1 (21.9%), presented technical difficulties in measuring the RRI.

Conclusion

FST is an economical and easily administered test to assess renal tubular function and can predict the occurrence and progression of S-AKI. RRI is a modest marker for predicting the need for RRT or persistent AKI.

## Introduction

Sepsis-associated acute kidney injury (S-AKI) is a frequently encountered complication in critically ill patients. Sepsis is found in about 40 to 50% of patients with acute kidney injury (AKI) in the ICU, leading to high healthcare costs and resulting in about four million deaths annually worldwide [1]. The mortality rates in S-AKI varied from 15-60%. Prevention of S-AKI is difficult because, by the time patients seek medical attention, most have already developed AKI. Thus, early recognition is crucial to provide supportive treatment and limit further insults [2].

Both Acute Kidney Injury Network (AKIN) and Kidney Diseases Improving Global Outcome (KDIGO) diagnostic criteria for acute kidney injury (AKI) include an increase in serum creatinine (S Cr) of >0.3 mg/dl (26.5 μmol/L) within 48 hours or an increase in S Cr to 1.5 times the baseline value, which is known or presumed to have occurred within seven days; or urine output < 0.5 ml/kg/hour for 6-12 hours [3].

There are several limitations to S Cr and urine output for the diagnosis of AKI. S Cr is not sensitive enough for early monitoring. It usually begins to rise days or weeks after the onset of AKI when the glomerular filtration rate (GFR) decreases by one-third to half, but not obvious in the diagnosis time window. In addition to the sepsis-induced reduction in muscle perfusion, which reduces creatinine production, the dilutional effects secondary to aggressive fluid resuscitation in septic shock lead to under-diagnosis of AKI when relied upon Sr creatinine levels [2].

Urine microscopy is also one of the conventional methods widely used to detect kidney disease. Patients with S-AKI had higher urine microscopy scores when compared with those with AKI from other causes. Urine microscopy was rather specific but poorly sensitive to detect worsening AKI [4,5].

Recently, various novel biomarkers have evoked interest among researchers in predicting the risk of AKI. JAMA Network, consensus statement 3 suggested a strong recommendation against using biomarkers for AKI risk assessment. However, future AKI biomarkers in patients with clinical risk stratification may help to detect those at risk of kidney complications after exposure. The negative predictive value is generally good, but the positive predictive value of most models is moderate to low. The use of biomarkers is limited by their availability, cost, variable cutoff levels, variations of sensitivity and specificity on the timing of measurement, reliability of single value and confounding by various co-morbidities and clinical conditions [6].

The furosemide stress test (FST) has been proposed to predict the need for renal replacement therapy (RRT) in patients with AKI, as it was found to be safe, feasible and well tolerated in critically ill patients. Further, FST has been demonstrated to be effective in predicting the severity of AKI [7].

Renal Doppler ultrasound can measure the renal resistive index (RRI), a sonographic index that reflects alterations in the blood flow profile of the intra-renal arcuate or inter-lobar arteries measured at the arcuate arteries (at the cortico-medullary junction). An increased RRI is often regarded as an indicator of renal vascular resistance. However, several non-renal hemodynamic parameters, such as vascular compliance, systemic pulse pressure, and heart rate and rhythm, seem to influence it [8]. Being a sonographic tool, it has the advantage of being a non-invasive, bedside tool devoid of the risk of radiation, and the test may be repeated any number of times. On the flip side, it needs the expertise to measure it accurately.

We are unaware of any previous study which examined the utility of RRI in predicting the risk of developing AKI. Thus, we planned this study to investigate the correlation of FST and RRI in S-AKI. The secondary objectives were to evaluate the effect of FST and RRI on the progression of S-AKI.

## Materials and methods

After approval from the institutional ethical committee (IEC No. 65/19), this prospective observational cohort study was conducted on patients admitted to the intensive care unit (ICU) of a tertiary care hospital & research centre from January 2020 to June 2021.

Sample size calculation

A previous study by Elsaegh et al. (2018) showed that FST had a sensitivity of 89.29 to predict outcomes in critically ill patients [9]. So, using the Buderer formula and the nomogram proposed by Malhotra and Indrayan (2010), with an anticipated sensitivity of 0.8 (80%), precision of 10% and a prevalence of 40% of AKI in patients of septic shock, 100 patients were needed to be recruited [10]. We decided to recruit 160 patients to provide for dropouts. Informed consent was obtained from the legal guardian of all recruited adult patients of either gender diagnosed with S-AKI.

Exclusion criteria included pregnancy, patients with pre-existing liver or kidney disease, allergies or known sensitivity to loop diuretics and evidence of volume depletion during furosemide administration. The baseline creatinine was estimated by the Modification of Diet in Renal Disease equation, assuming a low normal value for baseline GFR (75 mL/min/1.73 m^2^ of body surface area) [11]. All the patients were examined by an expert physician and supportive medical team through clinical and radiological examinations. In addition, the recruited patients underwent FST and calculation of RRI.

Furosemide stress test

The furosemide stress test (FST) is a provocative test used to distinguish patients with acute kidney injury (AKI) who are likely to progress to higher stages of AKI with the possibility of the need for RRT from those who are likely to have a less severe course. This test depends on demonstrating the two-hour urine output after a standardized high-dose FST in clinically euvolemic critically ill patients with sepsis to identify the severity and predict the progression of AKI. For furosemide-naive patients, an intravenous bolus dose of 1 mg/kg of furosemide was administered, and patients who had received furosemide within the past week were given a bolus dose of 1.5 mg/kg [7]. The urine output was then measured for the next two hours and based on the two hours' urine output, the patient was labelled as a responder (if urine output ≥200 ml) or non-responder (urine output ≤200 ml) to the FST. In addition, the patient's heart rate and blood pressure were monitored regularly, and urine output monitoring was continued for 24 hours.

The progression to AKI-KDIGO stage 3 (an increase in creatinine from baseline value by three times or creatinine ≥4 mg/dL or the initiation of RRT or a urine output < 0.3 mL/kg/hour for ≥24 hours or anuria for ≥12 hours) within 10 days of FST was studied as the primary outcome.

Calculation of renal resistive index (RRI)

Renal echography was performed by a radiologist/intensivist who was experienced in this technique, within the first 12 hours of admission and after restoring the hemodynamic status (by fluid bolus and/or inotropes, as per Surviving sepsis guidelines of 2021). Renal Doppler was performed on the interlobar arteries using a 2-5-MHz transducer. The Doppler gain was set to obtain a clear outline of flow waves with minimal background noise. The Doppler spectrum was optimal when at least three similar consecutive waveforms were visualized. The RRI was calculated as peak systolic velocity - end-diastolic velocity/peak systolic velocity. The RRI value is independent of the angle between the ultrasound beam and blood flow. Three measurements were performed on each kidney and averaged to obtain the mean RRI values for each kidney. If the difference in resistive indices (RI) between the two kidneys was less than 0.05, then the mean of the two values was noted as the RRI value for the patient. If the difference in RI was greater than 0.05, the patient was excluded because this difference could signify an acute unilateral ureteral obstruction.

The patients were grouped either into “progressors” (progression to AKI-KDIGO stage 3) or “non-progressors” (non-progression to AKI-KDIGO stage 3). The secondary outcomes were the composite end-point of achieving AKI-KDIGO stage 3 or death within 10 days of FST.

Demographic data like height, weight and gender, type of admission (sepsis or trauma), Simplified Acute Physiology Score II (SAPS-II), serum creatinine level, 24-hour urine output and calculated serum creatinine clearance (UV/P), MAP, heart rate, type and dose of catecholamine infusion, and the need for renal replacement therapy (RRT) were noted. Vital parameters at ICU discharge were also collected.

Statistical analysis

Data were analyzed using Statistical Package for Social Sciences (SPSS), version 23 (IBM Corp., Armonk, NY, USA). Discrete (categorical) data were summarised in proportions and percentages (%), while quantitative data were summarised as mean (SD), median or mode. Chi-square and other appropriate tests were used to check associations. In addition, a student t-test was used for group comparison. A p-value of <0.05 was considered statistically significant.

## Results

Of the 160 patients recruited, the data of four patients were not included in the final analysis because of inadequacy.

Table 1 shows the demographic details of the patients. There were more males (61.75%) than females (38.3%). Most patients were 40 years or below (53.9%).

**Table 1:** Demographic details of the patients

Demographic characteristics		N(%)
Gender, n (%)	Male	95(61.75)
	Female	59(38.3)
Age (years)	≤40	83(53.9)
	41-50	37(24.0)
	51-60	29(18.8)
	>60	5(3.2)

Table 2 shows the correlation between RRI on day 1 and the outcome (recovered, need for RRT and expired) during follow-up. Sixty percent of the patients who had RRI on day 1, less than 0.73, recovered, whereas only 22% of those who had RRI on day 1, greater than 0.73, recovered. Similarly, 46.6% of those with RRI at day 1, greater than 0.73, required RRT, compared to only 34.3% whose RRI at day 1 was less than 0.73. Moreover, 51.6% of patients with a mean RRI more than 0.73 expired, whereas only 35.5% of those with a mean RRI less than 0.73 expired.

**Table 2:** Correlation of result of FST (responder/non-responder) and RRI with various outcomes (recovered, needed RRT and expired) FST: Furosemide stress test, RRI: Renal resistive index.

Different Outcome	FST (Responder/Non-Responder)		RRI Day 1			Total
			Not done	<0.73	>0.73	
Recovered	FST	Responder	5	30	10	45
		Non-responder	4	0	1	5
	Total		9(18%)	30(60%)	11(22%)	50
RRT	FST	Responder	1	15	14	30
		Non-responder	13	10	20	43
	Total		14(19%)	25(34.3%)	34(46.6%	73
Expired	FST	Responder	0	2	6	8
		Non-responder	4	9	10	23
	Total		4(12.0%)	11(35.5%)	16(51/6%)	31
Total	FST	Responder	6	47	30	83
		Non-responder	21	19	31	71
	Total		27	66	61	154

Table 3 shows a statistically significant correlation between FST and RRI on days 1, 3, 6 and 9. Mean RRI was higher in the FST non-responder group than in the FST responder group on all days measurements taken.

**Table 3:** Association between Furosemide Stress Test and RRI on various days FST: Furosemide stress test, RRI: Renal resistive index.

FST	RRI (Mean±SD)			
	1^st^ Day	3^rd^ Day	6^th^ Day	9^th^ day
Responder	0.63±0.07	0.64±0.06	0.66±0.1	0.77±0.05
Non-responder	0.7±0.13	0.72±0.04	0.73±0.09	0.97±0.05
P-value	<0.001	<0.001	<0.001	0.005

Table 4 shows the cutoff values, sensitivities, specificities and accuracy of RRI and serum creatinine in predicting the need for renal replacement therapy based on the ROC curve.

**Table 4:** Cutoff values, sensitivities, specificities and accuracy of RRI and serum creatinine in predicting the need for renal replacement therapy based on the ROC curve RRI: Renal resistive index

Statistical parameter	RRI	Sr creatinine
Cutoff value	0.73	2.33 mg/dl
Sensitivity	35.1%	86.0%
Specificity	80.4%	57.7%
Accuracy	69.1%	89.7%

Table 5 shows the correlation of FST in patients with KDIGO stage 1 sepsis-induced AKI. It was observed that more patients among FST non-responders had progressed to KDIGO stage 3 (66.67%) from stage I compared to the FST responders (48%). This difference was statistically significant (p=0.01).

**Table 5:** Correlation of FST in KDIGO stage 1 and their progression during follow-up KDIGO: Kidney disease improving global outcomes, FST: Furosemide stress test.

KDIGO Stage on Day 1	FST	Non-progressor	Progressor		Total n(%)
		KDIGO 1 n(%)	KDIGO 2 n(%)	KDIGO 3 n(%)	
Stage 1	Responder	20(40)	6(12)	24(48)	50(32.46)
	Non-responder	2(13.3)	3(20)	10(66.67)	15(9.74)
	P-value	<0.001	0.689	0.011	

Table 6 shows the distribution of patients with technical difficulties encountered while calculating the RRI. Of the 154 patients recruited for the study, the examiners faced challenges while calculating the RRI in 32 patients. Of these, the highest number of patients had KDIGO stage 3 (50%), followed by stage 2 (28.1%) and stage 1 (21.9%).

**Table 6:** Analysis of technical difficulty in calculating RRI as a function of the KDIGO stage KDIGO: Kidney disease improving global outcomes.

KDIGO Stage	Technical difficulty		P-value
	No (n=122)	Yes (n=32)	
1	58(47.5%)	7(21.9%)	0.033
2	23(18.9%)	9(28.1%)	
3	46(33.6%)	16(50%)	

**Figure 1:**
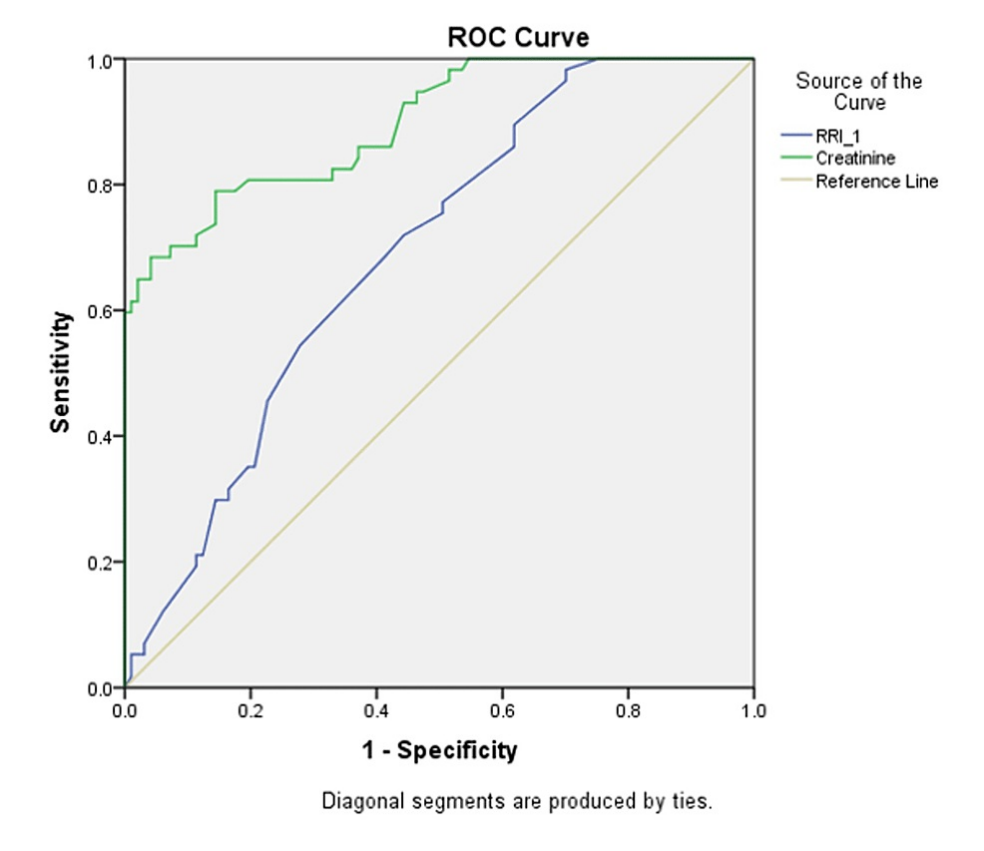
Receiver operating curve showing the sensitivity and specificity of RRI and serum creatinine. RRI: Renal resistive index

Figure 1 shows the receiver operating curve showing the sensitivity and specificity of RRI and serum creatinine.

## Discussion

The present study investigated the correlation of furosemide stress test (FST) and renal resistive index (RRI) in S-AKI and the effect of FST and RRI on the progression of severity of sepsis-induced AKI.

Furosemide is a loop diuretic which is not effectively filtered by the glomeruli. It is highly protein-bound and actively secreted into the tubular lumen through the human organic anion transporter system in the proximal tubule. Inside the tubule lumen, furosemide inhibits active chloride transport throughout the thick ascending limb of the loop of Henle. The reabsorption of sodium is inhibited, and there is resultant natriuresis which promotes increased urine flow. Chawla et al. proposed a validated and standardized approach to using furosemide to predict the progression of AKI to more advanced stages. They validated that a cutoff of 200 ml of urine produced at the end of two hours after furosemide administration had a sensitivity and specificity of 87.1% and 84.1%, respectively [7].

We observed that the need for RRT was more in the FST non-responders group compared to the FST responders group. The need for RRT in the FST non-responder group was highest in KDIGO stage 3 (71.9%), followed by stage 2 (43.9%) and stage 1 (27%), compared to the FST responder group. The difference between the groups of patients in these stages was statistically significant (p<0.001). Moreover, our data revealed that among patients who were FST non-responders on day 1, more of them progressed to KDIGO stage 2 (20%) and stage 3 (66.6%), compared to those among FST responders, where 12% and 48% progressed to stage 2 and 3, respectively (p=0.01). While 55% of patients with KDIGO stage 1 were FST responders, 52.2% of patients with KDIGO stage 3 were FST non-responders. Elsaegh et al. reported that the sensitivity and specificity of FST to predict the outcome of AKI were 89.29% and 93.75%, respectively [9]. Van der Voort et al. found that those patients who subsequently developed AKI had lower urine output at two hours following FST compared to those who did not develop AKI. They also showed that FST is valuable in predicting renal recovery following AKI, with an AUC of 0.84 [12].

Zhang et al., in a double-blind, prospective interventional cohort study involving critically ill patients with AKI, reported that FST non-responders were 3.79 times more likely to need continuous renal replacement therapy (CRRT) than FST responders. The area under the curve (AUC) for initiating CRRT was 0.966 (sensitivity 94.85% and specificity 98.04%, p<0.001). Thus, FST proved to be a safe and practical approach for predicting the initiation of CRRT in critically ill patients with AKI [13].

Renal resistive index (RRI) is a non-invasive Doppler-based parameter of intra-renal arterial resistance which can be performed repeatedly. The present study analyzed the correlation of RRI with outcome (recovery, need for RRT and mortality). The normal value of RRI was between 0.5-0.7. Although RRI helps predict the need for RRT, there is no unanimously accepted best cutoff value. We found that RRI was greater than 0.73 in those who needed RRT or expired. About 46.6% of patients with day 1 RRI value exceeding 0.73 required RRT, whereas 34.3% of patients with day 1 RRI < 0.73 needed RRT, but the difference was insignificant. RRI was higher in the group with KDIGO stages 2 and 3 than in the group with stage 1 AKI, but the difference was insignificant (p=0.16). Our study showed that the cutoff value of 0.73 had a sensitivity of 35.1% and specificity of 80.4% to predict the need for RRT (Table 4, Figure 1). The findings of our study agree with those of Darmon et al. [14]. A prospective multi-centric cohort study reported that the optimal cutoff of RRI for predicting persistent AKI was 0.71 with a sensitivity of 50% (CI 41-58%) and specificity of 68% (62-74%). The optimal value of RRI to predict the need for RRT was 0.73, with sensitivity of 51% (34-69%) and specificity of 77% (72-81%). RI is influenced by intra-renal arterial resistance, arterial compliance (e.g., renal interstitial and intra-abdominal pressures), age and central hemodynamic parameters [15]. Schnell et al., in a prospective double centre study involving 58 patients with severe sepsis or polytrauma, found that an RRI of greater than 0.707 on day 1 was predictive of the development of AKI stages 2 and 3 (by Acute Kidney Injury Network classification) with AUC of 0.91 and p-value (0.0004) [16]. Lerolle et al. demonstrated that a high RRI value of 0.74 on day 1 had a positive likelihood of a ratio of 3.3 for developing acute renal failure (according to RIFLE classification) on day 5 predicted AKI in patients of septic shock. The high renal arterial resistive index was associated with parenchymatous renal failure [17].

Limitations of the study

Arterial compliance is a critical parameter in determining RRI, which depends on renal vascular resistance. It is also influenced by renal interstitial, ureteral, and intra-abdominal pressure, possibly decreasing renal vascular compliance. RRI is also affected by asymmetrically affecting systolic and diastolic pressure and, thus, their velocities. RRI is also influenced by age and cardiovascular risk factors like diabetes mellitus, atherosclerosis and hypertension. The resultant reduced arterial compliance may raise the values of RRI even without AKI.

The present study is the first to report the incidence of technical difficulty encountered while calculating the RRI. The investigators faced technical challenges in 20.77% of the cases. Viazzi et al. described that a good-quality waveform may often be difficult to obtain and is influenced by several extra-renal causes [8]. We found that the highest number of patients of KDIGO stage 3 (50%), followed by stage 2 (28.1%) and stage 1 (21.9%), presented technical difficulties in measuring the RRI. Ours is the first study to report that calculating RRI was most challenging in patients with KDIGO stage.

## Conclusions

From the study findings, we conclude that FST is an economical and easily administered test to assess renal tubular function and can predict the occurrence and progression of AKI in patients with sepsis. RRI is a modest marker for predicting persistent AKI or need for RRT. The only disadvantage of FST is that it can be administered only once.
